# Exploring Perspectives on Antimicrobial Use in Livestock: A Mixed-Methods Study of UK Pig Farmers

**DOI:** 10.3389/fvets.2019.00257

**Published:** 2019-08-02

**Authors:** Lucy A. Coyne, Sophia M. Latham, Susan Dawson, Ian J. Donald, Richard B. Pearson, Rob F. Smith, Nicola J. Williams, Gina L. Pinchbeck

**Affiliations:** ^1^Department of Epidemiology and Population Health, Institute of Infection and Global Health, University of Liverpool, Neston, United Kingdom; ^2^Faculty of Health and Life Sciences, Institute of Veterinary Science, University of Liverpool, Neston, United Kingdom; ^3^Department of Psychological Sciences, Faculty of Health and Life Sciences, Institute of Population Health Science, University of Liverpool, Liverpool, United Kingdom; ^4^The George Pig Practice, Malmesbury, United Kingdom

**Keywords:** antimicrobial resistance, antimicrobial, antibiotic, antimicrobial use, farm animal, behavior, prescribing, mixed-methods

## Abstract

Increasing levels of antimicrobial resistance in human and veterinary medicine have raised concerns over the irresponsible use of antimicrobials. The role of administering antimicrobials in food producing animals most frequently falls to the farmer, therefore it is essential that their use of antimicrobials is both optimal and responsible. This study sought in-depth information on the drivers behind antimicrobial use behaviors and farmer attitudes to responsible use using a mixed-methodological approach. Initially, in-depth semi-structured interviews were conducted with a purposively selected sample of farmers (*n* = 22). A thematic analysis approach was taken to identify key themes from these qualitative data. The generalizability and variation of these themes was then tested on a larger randomly selected sample of pig farmers through a questionnaire study (*n* = 261). The influences behind antimicrobial use were complex with multiple drivers motivating decisions. There was no consensual opinion on what farming systems resulted in either a low or high antimicrobial requirement however, farmers reported that good management practices, low stocking densities, and a high health status were associated with low antimicrobial use. Farmers expressed desire to avoid the long-term use of in-feed antimicrobials, but identified barriers to discontinuing such behaviors, such as pig morbidity, mortality, and economic losses. The high cost of antimicrobials was described as a motivation toward seeking alternative methods of controlling disease to prophylactic use; however, this expense was balanced against the losses from an increased burden of disease. The high financial costs involved in pig production alongside the economic uncertainty of production and pressure from retailers, were identified as limiting the scope for improvements in pig accommodation and facilities which could reduce the antimicrobial requirements on farm. Long-term, sustainable and economically stable relationships between retailers and farmers may allow farmers to make necessary investments in improving management and housing in order to reduce antimicrobial use. Greater use and more widespread deployment of effective vaccinations were highlighted by farmers as being a feasible alternative to antimicrobial use in preventing disease.

## Introduction

There is increasing concern over the threat of antimicrobial resistance to human and animal health, with growing efforts by medical and veterinary professions to minimize prescribing and ensure that use is justifiable (1, 2). Antimicrobial use in livestock raises concerns over the potential public health implications from the transfer of resistant bacteria from animals to humans (3–5). A UK government commissioned review on antimicrobial resistance, led by Lord O'Neill, placed the livestock sectors under increasing pressure to collect baseline antimicrobial use data and to set species specific reduction targets (4). In the UK, the pig industry was found to have the highest antimicrobial use across the species sectors in 2015, with a baseline figure of 263.5 mg/PCU compared with the national cross-species figure of 57 mg/PCU (6, 7).

Antimicrobial use in pigs has been under the spotlight with the formation of working groups and research initiatives striving to address this high use and to promote responsible practices (8–10). Practices commonly employed in the pig sector such as the use of antimicrobials for disease prophylaxis and the commonality of the administration of in-feed antimicrobials (11–13) have been associated with high and indiscriminate antimicrobial use (4, 14). These behaviors coupled with the relatively high sales of antimicrobial products, authorized for use solely in pigs, have highlighted pigs as a priority species in the UK and Europe for gaining a better understanding of prescribing and use (15, 16).

There are diverse opinions held by both farmers and veterinary surgeons as to what behaviors are considered to be responsible and what routes should be taken to reduce indiscriminate antimicrobial use (17–20). Typically, most research has focused on the role of the veterinary surgeon in antimicrobial use decisions (21, 22), however, the role of actually administering antimicrobials typically falls to the farmer. Around 92% of UK pig production is overseen by a farm assurance scheme which require, as a minimum, a veterinary visit quarterly (23, 24). Thus, whilst the veterinary surgeon oversees the antimicrobial prescription or supply, and provides advice through a veterinary health plan, there is some freedom of choice with regards to antimicrobial use by UK pig farmers.

In human medicine, antimicrobial practices have been found to be motivated more by drivers relating to the social context of the prescribing environment such as managing time pressures, patient outcomes, relationships with patients, and a physicians' perceived role within the hospital than by concerns over antimicrobial resistance (25–28). Similarly, Bellet reported that drivers relating to the herd productivity, animal health, and welfare motivated anthelmintic use in dairy production; often to the detriment of considerations over anthelmintic resistance (29). Food producing animals occupy a unique position whereby animal management and the economic viability of a farm influence the antimicrobial use decisions of veterinary surgeons and farmers (19, 20, 26). Therefore, there is a need to explore antimicrobial use practices within the context of a pig farm.

The voluntary approach taken to antimicrobial use reduction in the UK coupled with the unique animal husbandry and management systems employed, place the UK pig sector in a unique position in comparison to other European countries. For example, around 40% of the UK breeding herds are raised on outdoor units, a feature which is particular to the UK, and is accompanied by diverse challenges compared to indoor breeding systems (30, 31). It is therefore essential that these and other drivers are explored further with farmers due to their direct effect on antimicrobial use on farms.

This study used a mixed-methods approach to describe pig farmers' antimicrobial use behaviors and explored attitudes to use in pig production in the UK. Thus, not only did the study describe what farmers reported to practically do, with regards to antimicrobial administration, but it also explored attitudes and perceptions to antimicrobial use behaviors. Consequently, the study was able to identify any mismatch between “desirable” behaviors, those are behaviors described in the guidelines as promoting prudent antimicrobial use, and “actual” behaviors reported by farmers. For example, an aspiration by the farmer to reduce antimicrobial use on the farm but barriers beyond their control limiting the scope to do so. The study builds on previous work which exploring veterinary surgeon perspectives to antimicrobial prescribing in the UK pig sector and focuses on the farmer as the end user (18, 22). At present, there are no published studies, which explore in-depth farmer perspectives on antimicrobial use in UK pig production, and as such, the study addresses a current knowledge gap. It is of particular importance due to the unique approach to both antimicrobial use policy and pig production taken in the UK.

## Methods

This study used a mixed-methods approach to explore UK pig farmers' perceptions on the balance between the costs and benefits of antimicrobial use in pigs. Individual semi-structured qualitative interviews allowed a more detailed exploration of farmer attitudes and perceptions around antimicrobial resistance and use; participants were free to discuss potentially emotive subjects on a one-to-one basis without the influence of other farmers (32). Subsequently, the themes were explored on a representative population of UK pig farmers to clarify themes, identify variation in the wider attitudes with regards to antimicrobial use in UK pig production.

### Participant Sample Population

The sample population was identified from the Department for the Environment and Rural Affairs (DEFRA) June Survey of Agriculture and Horticulture, 2011. A stratified random sampling methodology was employed to select participants based upon the type of farm, the number of sows/pigs on holdings. This sample was then further stratified by farm size with sampling proportional to the total number of pigs represented by that farm size group; such that large farms which represent the majority of pig numbers were not underrepresented (33) (Table S1). For the qualitative sample 150 farms were identified in England based on this sampling frame and farmers were invited to opt out from their telephone numbers being made available to the study. Existing contacts in the pig industry were used to sample farmers from Scotland however, Wales was excluded from the qualitative study due to the small number of commercial pig units. A purposive sampling approach was adopted to identify 22 participants as it enabled the identification and selection of information-rich participants for the qualitative interviews (34). Farmers were identified from a wide spectrum of farm types to ensure that the qualitative data encompassed knowledge and experience from across the pig sector (35).

For the questionnaire sample, 1,500 farms across England (92% of sample), Wales (<1% of sample), and Scotland (7% of sample) were selected using the aforementioned methodology (Tables S1, S2) to reflect the regional breakdown of the breeding herd (33). In order to avoid repetition, the questionnaire sample was distinct from the farms selected for the qualitative sample.

### Qualitative Methodology

Qualitative in-depth face-to-face semi-structured interviews were conducted with the pig farmers. An interview guide was designed based on a review of the literature, current issues surrounding antimicrobial resistance and results from focus groups previously conducted on the drivers of antimicrobial use in pigs (36) (Figure S1). The interview guide was constructed based on Lofland and Lofland's guide to preparing a qualitative interview (37). The interview guide provided key topic areas which were used to prompt and encourage farmers to express their views however, free conversation was actively encouraged. Open questions were used to encourage farmers to express their views around antimicrobial use in pigs. Interviews were undertaken by the author (LC) with an additional author (SL) also present for a number of interviews.

The interview audio recordings were transcribed verbatim, anonymized and the transcripts were transferred into Atlas.ti V.7.7.1 (Atlas.to Scientific Software Development) for data management. A theoretical approach to thematic analysis was used in which the coding of the transcripts were guided by the authors' pre-existing coding frame from an earlier focus group study (36). To ensure consistency in the analysis technique the approach described by Braun and Clark was adopted where the transcripts were read iteratively and coded data fragments were reviewed and classified to form minor themes (38). These minor themes were then further refined into major themes based on common subject areas. Themes were evaluated by a multi-disciplinary team to ensure that each was distinct, meaningful, and relevant to the research question (34). It was concluded that data saturation had been achieved when no new themes were defined from the interview transcripts and after that no further interviews were conducted.

### Questionnaire Methodology

The questionnaire content was based on the results from the qualitative interviews and consisted of the following four sections:

- Farm and participant information;
- Current opinion on antimicrobial use in pigs;
- Pig diseases and antimicrobial use on farm;
- Responsible antimicrobial use.

Open and closed questions were used with Likert scales to gauge opinion on agreement or importance. The questionnaire was created in Microsoft Word for postal distribution on 5 January 2015. A reminder postcard was sent to non-responders 3 weeks later and a second copy of the questionnaire was sent a further 3 weeks later to non-respondents.

Data were analyzed using Microsoft Excel 2010 (Microsoft Corporation, Redmond, Washington, USA) and SPSS Statistics 22.0 (IBM SPSS Statistics for Windows Version 22.0. Armonk, NY: IBM Corp). Descriptive statistics relating to the demographic information of respondents and their respective farms were produced and percentages determined for categorical response questions. Open questions were analyzed using a thematic approach. The open question responses were transferred into Atlas.ti V.7.7.1. (ATLAS.ti Scientific Software Development) for analysis. The free texts were re-read and the ideas generated were categorized and linked to form distinct codes. These codes described the thematic content of the data.

The study sought to explore the risk factors for antimicrobial use in the context of specific disease syndromes in pigs. Therefore, logistic regression analyses were used to determine drivers associated with antimicrobial treatment for specific disease syndromes on the respondents' farms in the year preceding the questionnaire study. Exploratory variables related to the pig density of the farm location, the housing and feeding characteristics of the farm, pig husbandry systems employed, the vaccination status of the herd, and the number of sows or pigs on the farm were assessed. Variable selection was based on risk factors for key disease syndromes identified by participants in the qualitative enquiry of this mixed-methods study.

Variables were assessed for each outcome using a Likelihood ratio (for categorical variables) or univariable logistic regression (for continuous variables) and any variables with *P* < 0.25 were tested for inclusion in multivariable models. The continuous variables (number of sows or pigs) were not normally distributed and were log-transformed in a natural log base 2 to compensate for the skewedness of these data. Therefore, the odds ratios were associated with a two-fold increase in the predicted variable. Models were built manually using a step-wise backwards elimination approach; the variable with the highest *P*-value was removed at each step. Two-way interactions of significant main effects were also tested. Variables were retained if their exclusion resulted in a likelihood ratio test statistic of *P* < 0.05 or if there was evidence of confounding.

### Ethical Approval

Ethical approval was granted from the University of Liverpool Veterinary Science Research Ethics Committee and the DEFRA survey control unit prior to commencing the study interviews.

## Results

### Interview Participants

A total of 22 interviews were completed with farmers from England and Scotland between April 2013 and March 2014. In the sample of 150 English farms, 30% of the farmers contacted over the telephone chose to opt out of the study. Forty-three participants from the remaining farmers were invited to take part in the study and 21 declined; reasons given included low staffing levels, a lack of time and harvest time. Therefore, 20 interviews were arranged within England using the database and a further two interviews were conducted in Scotland using existing contacts. Both of the farmers contacted in Scotland agreed to take part in the study. Interviews lasted between 30 and 90 min with an average length of 45 min. Demographic information on the farmers included in the questionnaire study are described in Table S3.

### Questionnaire Respondents

In total 511 (35%) participants responded however, only 261 of these were completed questionnaires (useable response rate was 18.1%); 250 were returned not completed or the questionnaire was returned to the researcher as the address was incorrect. The main reasons stated for non-response were farmers no longer keeping pigs (62%) or a duplicate listing of the same farm under two addresses (21.2%).

The majority of respondents were managers of a single unit (56%, *n* = 261) or multiple units (14%, *n* = 261), whilst 14% (*n* = 261) were independent farm owners. The majority of respondent farms (50%, *n* = 261) had only one member of staff, with 42% (*n* = 261) having two staff members and 8% (*n* = 261) having three or more. Farm managers oversee either independent farms or contract farms. The latter are owned and managed by a larger agribusiness (39). The questionnaire did not capture information on whether respondent farms were either independent or contract farms nor any information on the relationship of the farm with retailers or industry. There was wide variation in the number of pigs on farms with a median of 155 breeding sows and 1,150 feeding pigs on farms (Figure S1). The majority of the respondents worked on indoor units and only a small proportion of farms were classified as specialist with 4.6% (*n* = 259) of respondents being from organic farms and 1.9% (*n* = 259) being from specialist breeding units (Table S4).

### Overarching Themes

The study results revealed three major themes that influenced farmer attitudes with regards to antimicrobial use practices; farming systems; farm management strategies; and farm-level economics. These themes revealed a complex relationship between the farming system, quality of the farm management, and the antimicrobial requirements of the system. These major themes were not discrete and there was overlap between minor themes within them. For example, the economics involved in different farming systems is presented under the major theme of farming systems but is also an important contributor to the major theme of farm-level economics.

### Farming Systems

Farming systems were the most commonly discussed major theme across the qualitative interviews. This included all features relating to the farming system adopted such as husbandry practices, farm facilities, and biosecurity measures. Farmers frequently identified that farming systems had a major influence on the total amount of antimicrobial required on a farm. Additionally, farming systems were found to be related and linked with all of the major themes reported from the interview transcripts.

Farmers expressed strong but diverse opinions as to how farming, management and housing systems related to the health and welfare of pigs and consequently antimicrobial use. There was disagreement on what farming system participants' considered to be advantageous for the health of pigs; indoor or outdoor housing; slatted or straw-based pig accommodation. The majority noted that there were limitations and advantages to all production systems and that such contrasts were likely to result in a diverse range of disease conditions; with specific bacteria and viruses prevailing in some systems and being absent from others.

“*I think every system's got its strengths and its weaknesses, and every system exposes or isolates an animal from certain bacteria or virus[es]…”* (F004)

Unsurprisingly there was a tendency for participants to express more detailed opinions on farming systems that they were more familiar with. For example producers with experience of outdoor production predominantly considered that outdoor breeding herds were likely to have lower antimicrobial use when compared to herds housed indoors and often described the outdoor environment as a more natural system for the sow, which had a positive effect on their health and welfare.

“*I would say outdoor breeding is certainly a very, very low user of antibiotics… outdoors is a very natural system. The animal takes care of itself…”* (F018)

In contrast, a perception held solely by indoor producers expressed that sows and piglets on outdoor units may suffer negative health and welfare implications due to the extreme temperatures experienced.

“*… an outdoor pig, is not very happy in February. It's not covered by fur or feather. And it's not very happy in the summer when it's 80 degrees…”* (F005)

“*When you look at the weather we've had the last two winters, pigs have frozen to death outside in farrowing huts and drowned in farrowing huts.”* (F006)

An association between the farming system and the economics of production was identified by farmers. For example, whilst outdoor production was perceived by some as beneficial in minimizing antimicrobial requirements, farmers noted that the scope for outdoor production was limited as it was deemed less economically efficient. For example, outdoor was identified as producing fewer pigs per sow when compared with indoor systems. Additionally, participants expressed the opinion that intensive agriculture was necessary in order to produce enough meat to satisfy consumer demand.

“*I started off with outdoor pigs, and it works well, but you can't produce the number of pigs from an outdoor system as you can from a well-run indoor system.”* (F022)

“*The outdoor bred British pig isn't going to feed the world; in all honesty… it will be from intensive people.”* (F007)

The housing of feeding pigs on slatted floor systems sparked two opposing views amongst participants; some participants considered slats to be advantageous for pig health as they separated the animal from feces and urine. Conversely, the concept of the pig being housed above a slurry pit was not viewed to be a healthy environment for the pigs. In the following two examples participants used examples from human public health to justify their contrasting opinion.

“*The Victorians were the ones who back in the 19*^*th*^ *century separated the humans from muck, and brought sanitation, and that saw a huge reduction in disease… It's healthy for the pigs. It's more economical. One of the reasons it's more economical is the fact that we have to use less medicines, any in-feed/water, whatever.”* (F001)

“*The worst thing a pig does is get stuck in a confined area, with a fan environment, the standard way. They are sitting above a sewer. They sit on the slats above sewerage. Well yes, that's a very healthy way to live isn't it? Look at the trouble we had in London, in the early part of the century, with the Black Death and the plague and all the rest of it.”* (F008)

Low stocking densities and maintaining a high health status were associated with a low disease burden and minimal antimicrobial use by farmers. However, a minority of participants expressed concerns that a high health status herd may be vulnerable to novel disease due to an inherent immunological naivety to new pathogens.

“*If you want to reduce the drug usage in any livestock sector, reduce the stocking density, whether it is indoors or outdoors.”* (F010)

“*I think if you keep the health status up… it does cut your use of antibiotics markedly.”* (F014)

“*Health status… there is the potential… that everything is then that clean that you have had no pressure to a bug, and when something does come around, it knocks everything sideways.”* (F020)

**Figure 1:**
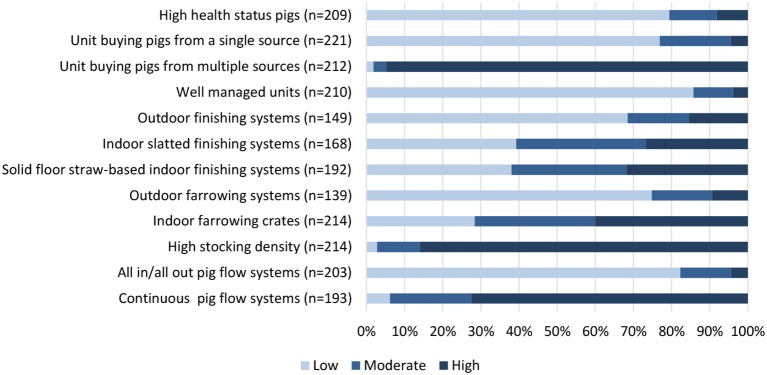
Questionnaire respondent opinion on the antimicrobial use requirements of different management features; low, moderate, or high.

Questionnaire respondents were asked their opinion on which management systems have the highest and lowest use of antimicrobials (Figure 1). The majority of respondents identified that high health status pig herds, systems sourcing pigs from a single source, well-managed units, and an all-in-all-out pig flow system were features associated with low antimicrobial use. Conversely, systems sourcing pigs from multiple sources, a continuous pig flow system and a high stocking density were linked with a high antimicrobial requirement. There was a spectrum of opinions with regards to whether outdoor or indoor systems have higher antimicrobial requirements although the majority of respondents shared the view that outdoor farrowing systems had a lower antimicrobial requirement when compared with indoor farrowing. In parallel with the qualitative results, opinion was divided between whether slatted or straw-based flooring systems were advantageous for minimizing antimicrobial requirements for pigs.

Exploration of farmer attitudes to management initiatives that would potentially drive a reduction in the total amount of antimicrobials used in the UK pig industry were explored in the questionnaire (Table 1). The results showed that there was widespread agreement amongst respondents that eradicating swine dysentery, modernizing pig accommodation, more effective and a wider range of vaccinations would be beneficial in reducing the total amount of antimicrobials used in pigs. Conversely, in parallel with the qualitative study, questionnaire respondents identified that the poor availability of highly skilled stock-people was a barrier to reducing total antimicrobial use in pigs.

**Table 1:** Questionnaire respondent attitudes to the role of management and economic drivers in reducing the total amount of antimicrobials used in the UK pig industry.

	**Barrier**	**Neutral**	**Beneficial**
**MANAGEMENT DRIVERS**			
Eradicating swine dysentery from the UK	11.0% (23)	8.0% (17)	81.0% (170)
Modernizing indoor pig accommodation	4.4% (9)	16.5% (34)	79.1% (163)
More effective vaccines	2.8% (6)	6.1% (13)	91.1% (195)
A wider range of vaccines	3.9% (8)	11.2% (23)	84.9% (174)
De-population and re-populating low health status pig herds with higher health status stock	2.5% (5)	18.5% (37)	79.0% (158)
Poor availability of highly skilled stock people	69.9% (137)	16.8% (33)	13.3% (26)
**ECONOMIC DRIVERS**			
Increased profitability of pig meat prices	4.6% (10)	26.9% (58)	68.5% (148)
Increasing the cost of antimicrobials for farmers	49.2% (103)	40.7% (85)	10.0% (21)
Decreasing the cost of antimicrobials for farmers	19.3% (39)	52.5% (106)	28.2% (57)
Reducing imports from other countries with high antimicrobial use	6.9% (15)	12.0% (26)	81.1% (176)
Prescription obtained from the vet and taken to a pharmacy to get antimicrobials (i.e., no longer sold by vet practices)	61.8% (126)	26.5% (54)	11.8% (24)

### Farm Management Strategies

The association between farming systems and antimicrobial use on farm was explored in greater detail through the questionnaire study. Firstly, farmers were asked about antimicrobial use on their farm in the year preceding the questionnaire and the conditions that antimicrobials were used for in different age categories of pigs. Lameness was reported to be the most common disease requiring antimicrobial treatment in both farrowing sows and dry sows, whilst gastrointestinal disease was most commonly reported in piglets and respiratory disease in feeding pigs (Table 2).

**Table 2:** Frequency of reported disease conditions requiring antimicrobial treatment in different groups of pigs on farms in the year preceding the questionnaire study.

	**Gastrointestinal disease**	**Respiratory disease**	**Reproductive disease**	**Lameness**
Farrowing sows	7.8% (9)	7.0% (8)	34.8% (40)	50.4% (58)
Piglets	56.5% (95)	17.3% (29)	0.0% (0)	26.2% (44)
Feeding pigs	22.6% (70)	44.1% (137)	0.6% (2)	32.6% (101)
Dry sows	0.0% (0)	7.4% (9)	17.4% (21)	75.2% (91)

Logistic regression analysis was used to explore the association between management features and whether a farm had used antimicrobials in the year preceding the questionnaire in each age category of pig; the final multivariable models are shown in Table 3 and univariable tables in Table S5. The farrowing sow and dry sow groups were more likely to have required antimicrobial treatment for lameness if they were housed on a farm with a greater number of sows. Conversely, dry sows were less likely to have required treatment with antimicrobials for lameness if they were housed in a closed herd in comparison to an open herd.

**Table 3:** Multivariable logistic regression analysis of respondent characteristics associated with requirements to use antimicrobials for different disease situations in different groups of pigs in the year preceding the questionnaire study.

		**No disease**	**Disease**	**Odds ratio**	**Lower 95% CI**	**Upper 95% CI**	**LRT *p*-value**
**LAMENESS IN FARROWING SOWS**							
Number of sows on farm (log base 2 transformed)	Median Minimum Maximum IQ range	105 2 40,000 338	320 2 4,000 624	1.3	1.1	1.4	<0.001
**LAMENESS IN DRY SOWS**							
Number of sows on farm (log base 2 transformed)	Median Minimum Maximum IQ range	40 2 40,000 270	285 10 7,000 630	1.5	1.3	1.8	<0.001
Closed herd	**No**	127 (67.2%)	62 (32.8%)	Ref			
	**Yes**	41 (58.6%)	29 (41.4%)	0.4	0.2	0.9	0.024
**RESPIRATORY DISEASE IN FEEDING PIGS**							
Number of pigs (log base 2 transformed)	Median Minimum Maximum IQ range	300 1 73,500 1,425	1,990 16 300,000 3,114	1.5	1.3	1.7	<0.001
Closed herd	No	80 (42.3%)	109 (57.7%)	Ref			
	Yes	42 (60%)	28 (40%)	0.38	0.2	0.8	0.006
Enzootic Pneumonia vaccination status	**No**	119 (50.6%)	116 (49.4%)	Ref			
	**Yes**	5 (19.2%)	21 (80.8%)	3.1	1.1	9.0	0.037
			21 (60%)				
**GASTROINTESTINAL DISEASE IN PIGLETS**							
Flooring type	Outdoor	33 (70.2%)	14 (29.8%)	Ref			
	Straw	35 (67.3%)	17 (32.7%)	5.2	2.1	13	
	Slatted	22 (33.3%)	44 (66.7%)	2.1	0.8	5.6	0.001
Sows PRRS vaccination status	No	156 (72.9%)	58 (27.1%)	Ref			
	Yes	10 (21.3%)	37 (78.7%)	5	2	12.1	<0.001

*LRT, likelihood ratio p-value*.

Feeding pigs were found to be more likely to have required antimicrobial treatment for respiratory disease if they were housed on a farm with a greater number of feeding pigs and if they were on farms with a vaccination programme for Enzootic Pneumonia. However, it is worth noting that only 10% (*n* = 261) of farms vaccination their feeding pigs against Enzootic Pneumonia. Feeding pigs were at a lower risk of having required antimicrobial treatment for respiratory disease if they were housed on a closed farm. Piglets housed on indoor farms on slatted or straw-based flooring were at a greater risk of requiring antimicrobial treatment for gastrointestinal disease when compared with piglets on outdoor units. In addition, piglets on farms with a vaccination programme for Porcine Reproductive and Respiratory Syndrome virus (PRRSv) in their sows were more likely to have required treatment for gastrointestinal disease compared to those from farms which do not have a vaccination programme in place. Only 18% (*n* = 261) of farms vaccinated their sows against PRRSv.

Antimicrobials belonging to the penicillin class were the most frequently used across all of the different categories of pigs accounting for 58.9% (*n* = 399) of recorded use. In sows and weaners antimicrobials belonging to the tetracycline class were the second most commonly reported class [13.5% (*n* = 104) and 15.7% (*n* = 127) of all recorded uses, respectively]. Whilst in piglets the fluoroquinolones [17.1 % (*n* = 82) of all recorded uses in piglets] and in finishers, the macrolides [22.1% (*n* = 86) of all recorded uses], were the second most commonly reported class. The overall reported frequency of use of the third and fourth generation cephalosporins was low (1.2%, *n* = 399) and use was only reported in piglets. Similarly, the polymixin group antimicrobial colistin was infrequently reported and only used in the piglet group (2.4%, *n* = 399).

The World Health Organization (WHO) classification for the highest priority critically important antimicrobial (HP-CIA) classes (40), as defined in 2011 (fluoroquinolones, third and fourth generation cephalosporins and macrolides), was discussed with study participants. During qualitative interviews farmers reported awareness of the concerns over the veterinary use of the HP-CIAs and felt strongly that that their use should be responsible.

“*There are several medicines that are not necessarily banned on-farm, are they, but they're restricted use because of the effect that that has had on human medicine, from what I understand.”* (F012)

In contrast, the questionnaire results showed that only 60.2% (*n* = 244) of respondents stated that they were aware of the issue of critically important antimicrobials. Of 122 farmers that attempted to identify critically important antimicrobials from a list of antimicrobials (including common trade names of products used in pigs), <50% of respondents were able to correctly identify HP-CIA products (Table 4).

**Table 4:** Antimicrobials identified as HP-CIAs by UK pig farmers (*n* = 122) from a provided list of drugs including both generic and trade names.

**Antimicrobial**	**Number of participants who identified antimicrobial as critical**
Amoxycillin (Amoxinsol, Stabox)	45 (36.89%)
Apramycin (Apralan)	12 (9.84%)
Ceftiofur (Excenel, Naxcel)^*^	30 (24.59%)
Colistin (Coliscour)	6 (4.92%)
Florfenicol (Nuflor Swine)	15 (12.30%)
Fluoroquinolones (Baytril, Marbocyl, Forcyl)^*^	52 (42.62%)
Lincomycin (Lincocin, Linco-spectin)	18 (14.75%)
Penicillin (Duphapen, Ultrapen LA)	50 (40.98%)
Spectinomycin (Spectam)	5 (4.10%)
Tetracyclines (Terramycin, Engemycin, Aurofac)	32 (26.23%)
Tiamulin (Denagard)	5 (4.10%)
Tilmicosin (Pulmotil)^*^	4 (3.28%)
Trimethoprim sulfate (Trimediazine, Tribrissen, Norodine 24)	9 (7.38%)
Tulathromycin (Draxxin)^*^	20 (16.39%)
Tylosin (Tylan)^*^	22 (18.03%)

* *Shows HP-CIA classes according to the WHO 2012 definition (40)*.

There was general agreement amongst farmers that the quality of the management system was a more important driver in the amount of antimicrobials used than the type of farming system employed; improving management practices was considered to be pivotal in reducing the antimicrobial requirements on a farm.

“*Any system can be badly managed. Half of the people that keep animals shouldn't be allowed; they should have a license to keep ‘bloody’ animals. Sorry, I get very cross about it… Management is a huge thing with managing antibiotic use.”* (F005).

Farmers suggested that a minority subset of farmers used antimicrobials in some circumstances as a “*management tool”* to compensate for a lack of re-investment in buildings and facilities. In these situations, interviewees felt that there may be improvements in animal husbandry and management systems that could replace the requirement for long-term antimicrobial use, however, these changes may be less economical than the use of medicated feed. This long-term or “*habitual”* use of antimicrobials was commonly cited by participants as an example of irresponsible use. Furthermore, a minority of farmers proposed that an outlying population of irresponsible farmers in some cases use long-term in-feed antimicrobials for their beneficial effects on growth rates in pig herds.

“*Because some farmers use antibiotics all the way through the finishers… It becomes a habit, I think, to use it. It becomes a crook… management-wise…”* (F011)

“*Antibiotics has become a prop for poor buildings and bad practice.”* (F016)

“*…a poorly managed farm, the chances are you will use more antibiotics than a well-managed farm. Of course there are always differences, you will get some guy who is very switched on, very well managed, and will use drugs as a growth promoter…”* (F017)

Half of the questionnaire respondents (50%, *n* = 118/234) identified that they had used antimicrobials on their farms for disease prevention and the majority of respondents reported that antimicrobial use for disease prevention and the use of in-feed formulations was either usually or always justified. In addition, most respondents agreed with the current policy that prohibits the use of antimicrobials for growth promotion, however 22.1% felt that such use was rarely justified whilst 7.2% felt that it was usually justified (Table 5).

**Table 5:** Questionnaire responses on the justification of antimicrobial use practices in UK pig production.

	**Never justified**	**Rarely justified**	**Usually justified**	**Always justified**
Antimicrobial use for treatment of pigs with disease	0.4% (1)	2.1% (5)	43.7% (104)	53.8% (128)
Antimicrobial use for disease prevention	18.9% (43)	29.8% (68)	44.3% (101)	7.0% (16)
Antimicrobial use for growth promotion	68.6% (151)	23.6% (52)	7.7% (17)	0.0% (0)
The use of in-feed antimicrobial formulations in pigs	17.6% (37)	25.2% (53)	48.6% (102)	8.6% (18)

Both methods identified that the decision over whether or not, and when, to withdraw prophylactic antimicrobials is a problematic one. Interviewees identified a fine balance between the economic cost of disease and antimicrobial costs; with the decision to discontinue medication being a compromise between the two.

“*…the cost of disease on any commercial unit… is huge. It comes down to what's your attitude in terms of risk and everything else? Sometimes the risk of breakdowns in health is such that… people are really, really reluctant to actually take it [in-feed antimicrobials] out.”* (F009)

The questionnaire explored drivers influencing the decision whether or not to withdraw prophylactic antimicrobials using an open question and free text box (responses are shown in Table 6). Clinical drivers such as the presence of disease on a farm, mortality rates and efficacy were the most common motivations for the continued use of in-feed antimicrobials whilst non-clinical drivers such as a reduction in herd performance, cost effectiveness, and veterinary advice were less commonly cited. In contrast, the decision to discontinue in-feed antimicrobials was predominantly driven by non-clinical features such as high cost, veterinary advice, and concerns over antimicrobial resistance. Highly skilled staff were identified as an integral part of a well-managed pig unit. Some participants directly linked the quality of staff skills with antimicrobial use. For example, poorly skilled staff were considered to be a limitation in reducing use on some units as stock people who are disinterested and less skilled in their work may use antimicrobials as a short-term solution to a longer term problem.

“*Good stockmen are worth their weight in gold… If you're not interested and you're not bothered, what's easier than chucking a load of antibiotic food in? It makes it right for the short term, doesn't it?”* (F006)

In contrast, farmers defined good stock people as those with an innate skill and ability to detect any discomfort in the pig herd before it became a major problem.

“*So the sharper the stock man, the more effective you can deal with issues before it gets out of hand, and make decisions fast in terms of segregation or that sort of thing.”* (F013)

The recruitment and retention of highly qualified staff was problematic; they identified that a lack of availability of highly motivated staff was a pressure on the pig industry.

“*The biggest problem we have as an industry is finding good staff… everybody I talk to is struggling to find people, who want to actually spend time with animals, let alone, are happy to work seven days a week, you know. But that's what animals have to have, a seven day a week commitment.”* (F008).

**Table 6:** Themes volunteered by questionnaire respondents as influencing the decision to continue or discontinue in-feed antimicrobials on their farm.

**Drivers identified by farmers as influencing the decision to continue in-feed antimicrobials on their farm**			**Drivers identified by farmers as influencing the decision to discontinue in-feed antimicrobials on their farm**		
	***n***	**Example quotation**		***n***	**Example quotation**
Known disease issues in pigs	21	“*Had an underlying problem on farm and needed it cleared up”*	High cost	31	“*Can be costly if there are feed spillages and if over treating all pigs instead of injecting 10–20 instead.”*
Veterinary advice	21	“*Only if vet considers this wise”*	Improvements in pig health	19	“*The disease burden has reduced”*
To prevent a reduction in herd performance	16	“*Improved performance of pigs”*	Discontinue use when clinical signs no longer present	17	“*If clinical signs have disappeared or receded to us having to confidence to stop feed medication.”*
Prevention of disease is better than treating disease once clinical signs are apparent	13	“*Prevention always better. Especially weaning—time of most stress”*	Veterinary advice is to discontinue in-feed antimicrobials	15	“*The vet decides about when to start and stop in feed antibiotics”*
Good efficacy	11	“*We used to because it was easy and effective”*	Ineffective if used long term	9	“*Ineffective if used too often”*
Disease problems occur if in-feed is withdrawn	9	“*Having tried to withdraw antibiotic, disease re-establishes”*	Concerns over antimicrobial resistance	7	“*It helps cause resistance and it is no longer a responsible option to use them long term.”*
To maintain a high level of welfare	8	“*Welfare of pig. If stop animal may break down”*	Improvements in weather conditions	6	“*The weather and time of year is a major factor. Pigs can be moved away on another site in summer months so shed can be washed, rested and re-furbed if needed.”*
Cost effective to continue with medication	8	“*Prevention has good cost/benefits”*	Personal concern over the ethics of the long-term use of in-feed antimicrobials	5	“*The feeling that things have changed”*
To prevent high mortality rates	6	“*Insurance against unforeseen losses especially if disease is causing no? deaths.”*	Industry pressure to discontinue use of in-feed antimicrobials	5	“*Some companies have routinely stopped in feed to impress retailers”*
Respiratory disease problems	5	“*All of the pigs are coughing”*			
Time of year when disease is common	5	“*There are certain times of year that is unwise to stop, you stop in spring when environment is on your side.”*			

### Farm-Level Economics

Farmers focused on farm-level cost effectiveness and profitability when considering the economic drivers behind antimicrobial use decisions. A more detailed discussion of the wider economic aspects of antimicrobial use in pig production, such as food supply chains and the pharmaceutical industry, were beyond the scope of this study and were not concerns volunteered by farmers through the qualitative interviews. The high financial costs involved in pig production, juxtaposed with the economic uncertainty of production, were identified as limiting the scope for improvements in pig accommodation and facilities which could reduce the antimicrobial requirements on farm. Farmers expressed a desire to minimize the economic burden from disease and associated the absence of disease with low antimicrobial use and thus reduced veterinary costs. This concept echoes the importance placed by farmers on good management for minimizing antimicrobial use.

“*…you cannot run a pig farm profitably with high levels of endemic disease.”* (F009)

Economic pressure was considered by some to limit the scope to reduce antimicrobial use on farm. Whilst many farmers described an aspiration to reduce antimicrobial use, a “desirable behavior,” the high cost of re-investing in housing or facilities was identified as a barrier to behavior change.

“*…accommodation is a key part of improving health, we then need to be able to be reinvesting in quality finishing accommodation. And you need a desire to be able to reinvest the money. So you need some profit to start with.”* (F001)

Farmers considered that the high cost of antimicrobials was a motivation toward ensuring that their use was minimal on farms.

“*…there are huge cost implications with antibiotics… So we're obviously all the while looking to see, “Do we need that in the feed, that antibiotic?” But then equally you look and say, “Well if we don't have it in there, what's the cost of that going to be?…at the end of the day, we're running a business here trying to produce meat for people to eat.””* (F004)

Many identified that such costs acted as an incentive to seek alternative therapeutic and prophylactic methods to antimicrobial use. For example, farmers proposed that the introduction of a vaccination protocol to prevent disease alongside achieving and maintaining a high health status could minimize the costs of antimicrobials and offer farmers greater profitability from the pig herd.

“*If you can stabilise health and you can manage that health, then, you certainly will be using a lot less reactive-type drugs if you can have good health plans, and have good vaccination programmes with preventative use of those drugs. Then, you should be using less and you should have a more profitable unit, without doubt.”* (F022)

Farmers' described that this desire to minimize antimicrobial costs was founded on the substantial economic pressure on the pig industry to produce pigs at a low cost to the consumer; the majority identified this as a long-term pressure from retailers. Discussion over retailer pressure was emotional and sparked passionate opinions.

“*At the end of the day we are really pressurising them [stock people] to reduce costs, so we don't want to use medication unless we have to. We would rather do the testing, we would rather use vaccines.”* (F018)

“*Continual supermarket pressure in terms of not paying the right price for the product. Also the feed costs have been ridiculous these last few years… the financial pressure on pig farmers has been extraordinary.”* (F013)

The majority of interviewees felt that “decoupling” prescribing and dispensing, such that veterinary surgeons are no longer able to sell antimicrobials, would have little effect on overall antimicrobial use. However, a minority felt that this may be a beneficial intervention to reduce antimicrobial use by some irresponsible veterinary surgeons; who may be driven to prescribe by the ability to profit from antimicrobial sales.

“*I could see why in the market, there could be an incentive for them to over-prescribe because there was a profit incentive. I would like to think that the vets are responsible enough not to do that, but I could see why, potentially, it could be an issue, and I could see why some countries have split the different services.”* (F014)

Questionnaire respondents identified that farm economics and antimicrobial costs could play a role in reducing the total amount of antimicrobials used in the UK pig industry (Table 1). There was shared agreement amongst the majority of farmers that increased profitability in pig meat prices and reducing importation from high antimicrobial use countries would be beneficial in reducing the total amount of antimicrobials used in pigs. There was a range of opinions on the effects should the cost of antimicrobials be increased or decreased for farmers, however, the majority felt that it would have little effect on total antimicrobial use in pigs.

The majority of farmers were unsure on the future sustainability of the UK pig industry, an opinion founded on uncertainty over the economic viability of pig production. Whilst farmers considered that pig production had the potential to produce meat at low costs they expressed concerns that the low prices paid by retailers were hurdles to the profitability of pig enterprises. Some participants depicted a cyclical economic landscape in pig production whereby the industry continued going through phases of both growth and decline. However, farmer opinion was divided between those optimistic and those pessimistic as to whether the future would be toward the financial rewards phase of the cycle. Most participants considered that the retailers and associated consumer demands would influence the future and sustainability of the sector.

“*We are back to the supermarket actually putting their money where their mouth is by continuing to source UK pigs, and because of our regulation, it costs more.”* (F020)

“*I have a mildly optimistic view, mainly because I think the levels it's at the moment are, historically, as low as they've ever been since we developed a pig industry…We've never been self-sufficient in pig meat. I just think the potential's there…Beef and sheep are going to be too expensive. Pig meat can still be produced economically, so I think it has brilliant potential. The rest of Europe eats twice as much as we do.”* (F012)

“*The pig industry, in its cycle, is always moving from – I'd like to say boom to bust, but we don't have much boom, and it's generally bust.”* (F001).

## Discussion

The study a mixed-methodological approach to identify farmers' perspectives on antimicrobial use behaviors in pig production in the UK and to explore potential routes to antimicrobial use reduction. Farmers described an economic benefit to antimicrobial use in terms of reducing the disease burden on farm, however, this was balanced against the high cost of antimicrobials and a drive amongst farmers to seek alternative methods of preventing disease to antimicrobial use. Farmers held a spectrum of opinions as to the antimicrobial requirements of different management systems; however, there was agreement that good management was key to reducing antimicrobial requirements.

In agreement, the literature highlights that the quality of the management is essential in minimizing the antimicrobial requirements of a farm with farmers describing the importance of an optimal environment for pigs (13, 31, 41, 42). Many identified that a lack of economic certainty had resulted in the inability of many farmers to reinvest in the housing and management improvements needed to reduce their reliance on antimicrobials. Such conflicts are recognized in other studies with Stevens et al. (31) reporting that farmers' who felt that their farm environment could be improved used more in-feed antimicrobials compared to those that did not perceive that improvements were necessary. Similarly, Alarcon et al. (43) highlighted that farmers recognized a need to balance the high cost of disease with augmenting production costs (31, 43).

The adoption of herd management strategies and improved biosecurity may be a more cost-effective and feasible alternative to preventing disease than routine antimicrobial use (44, 45). The most important driver of implementing such measures, or changing behavior, in pig farmers is the potential economic rewards in profitability and reducing antimicrobial costs (19). However, economic uncertainty, fluctuating prices and increasing retailer demands put farmers under increasing financial pressure (46). Farmers cited the unpredictable and downward price trends from retailers as being responsible for the economic instability they had experienced. This has been described as a concern for farmers and veterinary surgeons in the pig sector (43, 47). However, in contrast retailers have been identified as actors in promoting minimal and responsible antimicrobial use behaviors in pig producers (10). Long-term, sustainable, and economically stable relationships between retailers and farmers may allow farmers to make necessary investments in improving management and housing in order to reduce antimicrobial use. For example, offering economic rewards for low use may incentivize farmers to engage in seeking alternatives to antimicrobials and to optimize use.

It has been proposed that the ability to profit from the sale of antimicrobials may act to incentivize overprescribing in veterinary surgeons (48–50). Whilst the majority of farmers felt that this would not motivate prescribing by most veterinary surgeons a minority, felt that it may drive prescribing by some veterinary surgeons. In agreement, Visschers et al. found that farmers perceived that “decoupling” would have little importance in reducing antimicrobial use in pigs (20) whilst Postma et al. reported that veterinary surgeons felt that retaining the right to sell antimicrobials was a motivation to reducing antimicrobial use (21).

The outcomes from “decoupling” policies are diverse across the countries that have introduced such legislation; ranging from Norway and Sweden, with some of the lowest sales to Italy, with one of the highest (51). The importance of antimicrobial sales for a veterinary practice is highly variable and depends on the relationship of the practice pharmaceutical suppliers and the costing structure of the practice. For example, profit from the sale of antimicrobials often subsidies the costs of veterinary visits for farmers (52). Consequently, any such policy to regulate antimicrobial sales may have wider impacts on the structure of practices, costs of veterinary services for farmers, practice profitability, and on the veterinary surgeon-farmer relationship.

Responsibility for the prudent use of the HP-CIAs in livestock is shared between the veterinary surgeon, as the prescriber and the farmer, as the end user. Thus, there is a need for farmers to be aware of concerns over their use (53) and this is of particular importance with an increasing move from retailers to introduce antimicrobial use policies, which regulate the use of antimicrobials. For example, dairy farmers who are members of the Tesco Sustainable Dairy Group are required to reduce their use of HP-CIAs and to provide antimicrobial susceptibility test results to support any usage on farm (54). Similarly, there has been a move in some countries for retailers to market meat as “raised without antibiotics” in response to growing concerns over antimicrobial use in livestock (55, 56). Therefore, it is important that farmers have an understanding of the HP-CIA classes and concerns about their use in livestock.

Whilst knowledge of the public health concerns over the HP-CIAs are reported as being widespread amongst veterinary surgeons (57), there is no published literature which explores farmer awareness of the issue. All interviewees expressed awareness of the HP-CIAs, however only 60% of questionnaire respondents reported awareness of the issue and less than half of these could correctly identify HP-CIAs from a list of commonly used antimicrobials. This mismatch in participant awareness, despite being drawn from the same sampling frame, may reflect that interviewees consenting to face-to-face interview were more likely to have a pre-existing interest in antimicrobial use and resistance and thus may be more likely to be aware of HP-CIAs. Since this study was conducted there have been numerous education initiatives to raise awareness of antimicrobial resistance and HP-CIA use amongst farmers with the aim of reducing HP-CIA use, alongside overall use (7, 54, 58, 59). In addition, the Pig Veterinary Society published guidelines advising that HP-CIAs should not be used as first line antimicrobial options (60). Thus, the increased communication, from key stakeholders on the importance of prudent use of HP-CIAs has hopefully resulted in greater knowledge on the issue by UK pig farmers since the completion of this study, however this should be reassessed.

The prophylactic use of antimicrobials at group level has been identified as a frequent behavior in European pig production (26, 31, 61, 62), in spite of pressure by the European Parliament to restrict the practice (63). In response there has been a move to evaluate alternative methods of preventing disease (10, 19, 44); a concept desired and favored by farmers in this study. Similarly, other studies have associated the long-term use of in-feed antimicrobials with irresponsible use behaviors (20, 64). However, in parallel with the opinion reported in the literature (11, 18, 26, 31, 36), participants felt that the use of antimicrobials for disease prophylaxis was justified in some circumstances. In contrast, a minority expressed concern that there may be some irresponsible pig producers who use antimicrobials as a long-term “management tool” in place of husbandry improvements.

The decision over whether to continue or withdraw prophylactic medication was problematic for farmers due to the unpredictable nature of disease and the potential costs should disease return on the discontinuation of antimicrobials. These are common concerns amongst pig veterinary surgeons and farmers (47, 64). The importance farmers placed on the cost-effectiveness of these decisions is also shown in a study which identified that economic considerations were crucial in pig farmer decisions on disease control (43). The Pig Veterinary Society advise that the need for prophylactic antimicrobials should be reviewed at quarterly farm assurance visits and this should form the basis for responsible antimicrobial use (65). Further guidance directed at farmers and veterinary surgeons on the importance of reviewing preventive antimicrobials and alternative methods of preventing disease would allow more informed decisions to be made with regards to antimicrobial use for disease prevention.

Highly skilled stock people were perceived by farmers to be an essential component of a well-managed pig unit enabling early disease recognition and prompt antimicrobial treatment. In parallel with the literature farmers reported that not all stock people possess these essential skills (26, 66, 67). Fertner et al. (41) reported that highly skilled staff were better able to identify disease signs early, however, the study reported that veterinary surgeons did not necessarily correlate this with low antimicrobial use (41). This study also highlighted the importance of spending sufficient hours observing pigs in order to recognize any issues in a herd. In other studies the presence of highly-skilled stock people, who show empathy for pigs under their care, has been correlated with positive health, welfare, and productivity parameters in pigs (68–70). Thus, there is a potential for structured education and training for stock people on pig herd health management with a focus on responsible antimicrobial use.

Lameness has been identified as a major driver toward antimicrobial use in sows and is one of the most significant reasons for both euthanasia and early culling in breeding pigs (31, 71). It is of great economic importance to pig production due to its negative effects on sow fertility and herd productivity (72), and the study results identify it as the most important clinical indication for antimicrobial use in sows. Respondents reported sows from herds with a greater number of sows were more likely to require antimicrobial treatment for lameness. The literature reveals contrasting results with some studies reporting that an increase in herd size is associated with a decreased risk for the development of lameness (71, 73) whilst, Willgert et al. notes that factors associated with larger and more productive herds pose an increased risk for lameness in the English pig herd (74). Interpretation of these results need to be considered within the specific context of the study as the findings assess the use of antimicrobials for lameness. Therefore, it may be that stockpersons on larger pig units are more likely to identify and treat lameness, or are more likely to have a proactive prevention plan for lameness and may have better handling facilities to treat lameness when compared with smaller pig herds. Presently there is a knowledge gap with regards to risk factors for lameness in sows which an area which warrants further research.

Respiratory disease was found to be the most important disease syndrome requiring antimicrobials in feeding pigs (31). Conversely, in piglets gastrointestinal disease was more common. Additionally, these were the most frequently reported conditions that required antimicrobial treatment in all groups of pigs across Europe (75). In parallel with the findings for lameness in sows the study results revealed that feeding pigs from herds with a greater number of pigs were more likely to require treatment for respiratory disease. Similarly, the literature identifies that a larger herd size presents a greater risk for respiratory disease when compared with smaller herds (76–78). The policy of maintaining a closed herd, whereby no new animals are introduced, has been associated with improved animal health and productivity as well as lower antimicrobial use (42, 79–81). In agreement, the study revealed that the risk of requiring antimicrobial treatment for lameness in dry sows and respiratory disease in feeding pigs was lower in closed herds when compared to those that were open.

Respondents identified that piglets housed outdoors were less likely to have required antimicrobial treatment for gastrointestinal disease in comparison to piglets housed indoors on a slatted or solid-floor with straw bedding. There are very few studies which explore the relationship between disease status, antimicrobial use and outdoor or indoor production systems, however, Stevens et al. (31) concluded that overall, for all disease conditions, outdoor breeding units spent significantly less on injectable antimicrobials for pigs when compared with indoor breeding (31). A study by Kilbride et al. into pre-weaning piglet mortality found that diarrhea was a more frequent cause of mortality in piglets housed indoors when compared to those reared outdoors (82). However, *Salmonella*, a significant cause of diarrhea in the UK pig herd (83), has been shown to have a higher incidence on outdoor units when compared with indoor farms (84, 85).These findings may reflect that both internal and external biosecurity are easier to implement and maintain on an indoor unit when compared to an outdoor herd (86). In order to fully understand the risks for pre-weaning diarrhea, and the need for antimicrobial treatment in piglets further research into the effects of environment, such housing systems and flooring types is needed. In addition, work to identify and describe effective biosecurity measures to prevent the introduction, or spread, of diarrheal pathogens for indoor and outdoor systems is essential.

Vaccination programmes are used to improve the immunity of pigs, reducing the risk of clinical signs of disease, and consequently reducing the need for antimicrobial treatment. Thus, vaccinations are reported to be an alternative method of controlling disease to antimicrobial use (44, 80, 87). In agreement, questionnaire respondents felt that the availability of more effective vaccinations and a wider range of vaccines would be beneficial in reducing overall antimicrobial use. In addition, interviewees defined vaccination as a feasible alternative route for preventing disease to antimicrobial use and is an area where further research is needed.

The results from the logistic regression analysis contrast with the general principle of a vaccination, as protective against disease, as the study found that having a vaccination programme was associated with an increased use of antimicrobials on farms. This contradiction has been observed in other studies exploring the relationship between antimicrobial use and vaccination in pigs (80, 88). These results may represent the attitudes of farmers or their veterinary surgeons, that using a greater number of vaccinations and antimicrobials is a more effective insurance against disease than using fewer pharmaceutical products (19, 26, 80). Alternatively, these contrasts may reflect that pig herds with vaccination programmes have a higher disease pressure than herds without vaccination programmes and that in vaccinated herds disease is yet to be controlled through vaccination alone. Thus, such farms may be relying on a combination of vaccination and antimicrobials in order to control the clinical signs of disease (80). Furthermore, the results from this study should be considered in relation to the small respondent population that had a vaccination programme in place. Further research to determine the true advantages of vaccination in terms of reducing antimicrobial use is required. This work should include a detailed exploration of the farm-level vaccination programmes including information on the vaccination types used, history of disease pressures encountered on farms and the indications for antimicrobial treatment in pigs.

The adoption of mixed methods acted to combine the strengths of both qualitative and quantitative enquiry to increase the depth and breadth of the understanding of farmers' perceptions on antimicrobial use and how this affected their use behaviors (89). This provided a more complete picture of perceptions and beliefs than either method could have done individually (90–92). For example although interviewees expressed awareness of HP-CIAs, when tested further this was not consistent across the larger population and less than half of questionnaire respondents were able to correctly identify HP-CIA classes from a list of antimicrobial active ingredients.

The study presented an overview of farmers' attitudes to antimicrobial use and as such did not provide a detailed analysis of how respondent demographics may influence antimicrobial use behaviors. With the adoption of a mixed-methods approach, it was beyond the scope of the study to undertake a more detailed statistical analysis of questionnaire responses; such as those seen in purely questionnaire studies into antimicrobial use practices (20, 22, 93). In addition, the questionnaire content focused on findings from the qualitative study and the contrasts between the farming systems, for example, the differences between indoor and outdoor production, opposed to investigating differences between the characteristics of respondent farmers. Therefore, these results provide a baseline of information on farmer attitudes to antimicrobial use in the pig sector as a whole, which warrants further exploration with regards to how respondent characteristics influence antimicrobial use behaviors and attitudes to use.

Although the useable response rate for the questionnaire study was only 18%; overall 35% of the questionnaires were returned, but 62% of those returned were from respondents who were not eligible to be included in the study; most frequently because they no longer kept pigs. The low response rate may have introduced bias as the responders may be different in terms of antimicrobial use and perceptions, to non-responders. Potential reasons for non-response across both the qualitative interviews and questionnaire may be related to the sensitivity of the issue of antimicrobial use in pigs. There has been increasing pressure from the general public, politics, and media regarding antimicrobial use in food producing animals and it is possible that this scrutiny may have resulted in a reluctance for farmers to discuss their current practices for fear of negative consequences (26, 94, 95). In addition, there may be limitations to self-reported behaviors with participants responding to questions in the manner in which they perceive is expected (96, 97), thus, there is a potential in this study that respondents may report antimicrobial use behaviors that they consider are optimal and responsible rather than their actual practices. However, the very open and honest discussion in the qualitative interviews, including discussions on highly emotive subjects such as the potential public health consequences from antimicrobial use in pigs, suggest that the study presents accurate perceptions and behaviors (98).

## Conclusions

Farm profitability and disease burden were reported to be precariously balanced; with farmers identifying that costs and benefits were major drivers in antimicrobial use decisions. Farmers identified that improving management practices and stabilizing prices would be routes through which antimicrobial use can be minimized in the UK pig sector. Further research is needed to identify cost-effective management strategies to reduce antimicrobial use in typical UK production systems.

Antimicrobial use for disease prophylaxis remains an important disease management tool for many producers however, farmers reported a need to seek alternative methods for disease prevention. Providing detailed guidance on reviewing routine preventative antimicrobial use and alternative methods for disease prevention would allow more farmers to make informed decisions on antimicrobial use.

## Data Availability

All datasets generated for this study are included in the manuscript and/or the Supplementary Files.

## Ethics Statement

Ethical approval was granted from the University of Liverpool Veterinary Science Research Ethics Committee and the DEFRA survey control unit prior to commencing the study interviews.

## Author Contributions

GP, NW, SL, and SD conceived the study. LC was responsible for drafting the manuscript, undertaking the qualitative interviews along with SL, designing and circulating the questionnaire and analyzing the data. GP, RS, and RP oversaw the questionnaire design. GP assisted with statistical analysis. SL, GP, and ID oversaw the qualitative interview study design and analysis of data. RP and RS provided specialist clinical advice to ensure that the study was clinically relevant. All coauthors played a significant role in the editing and reviewing of this paper.

### Conflict of Interest Statement

RP was employed as a specialist pig veterinary surgeon by The George Veterinary Group which is a private veterinary practice. The remaining authors declare that the research was conducted in the absence of any commercial or financial relationships that could be construed as a potential conflict of interest.
