# Supplementary material for: Exploring Perspectives on Antimicrobial Use in Livestock: A Mixed-Methods Study of UK Pig Farmers
Source: Front Vet Sci. 2019 Aug 2;6:257. doi: 10.3389/fvets.2019.00257 (PMC6688534; doi:10.3389/fvets.2019.00257)
Supplement: Supplementary file 1 [file Data_Sheet_1.PDF]

Supplementary Material

1 Supplementary Tables

1.1 Table S1

Sampling frame for the questionnaire study based on sampling the farms proportional to the total number of pigs represented by that farm size group (such that large farms that represent the majority of pig numbers in UK were not underrepresented) stratified by holdings with female breeding and fattening pigs, fattening pigs only and breeding sows only.

**Type of farm**	**The number of sows/pigs on holdings**		
**Holdings with both female breeding and fattening pigs (60%)**	**5-24 female sow breeding herd**	**25-99 female sow breeding herd**	**100 and over female sow breeding herd**
**Percentage for sample**	4%	8%	88%
**Fattening-only holdings (50%)**	50-299 fattening pigs	300-999 fattening pigs	1000 and over fattening pigs
**Percentage for sample**	3%	17%	80%
**Breeding-only holdings (10%)**	5-24 female sow breeding herd	25 to 99 female sow breeding herd	100 and over female sow breeding herd
**Percentage for sample**	7%	7%	86%

(Source – Defra, 2013)

1.2 Table S2.

Regional breakdown of pigs per thousand head in the UK by region and the proportion of the farms required in the sample population from each region.

Region	Thousand head pigs	% of total	Questionnaire study sample	Qualitative interview sample
**England**	4066	92%	1380	18
**Scotland**	314	7%	105	2
**Wales**	25	1%	15	0
**Total**	4405	100%	1500	20

(Source – Defra, 2013)

1.3 Table S3.

Demographic data for farmers in a qualitative interview study into antimicrobial use behaviours in UK pig production (n=22).

**Interview ID**	**Gender (M/F)**	**Manager or owner**	**Farm type - farrow-to-finish (FTF), breeding only (BO), finishing only (FO)**	**Farm type – independent or company**	**Housing type – indoor or outdoor, slatted or straw-based**	**Number of sows**	**Number of pigs**	**Producing under any assurance schemes**
**F001**	M	Manager	FTF on multi-site production	Company	Slatted indoor	7000	Progeny	Red Tractor
**F002**	M	Manager	FTF	Company	Slatted indoor	3000	Progeny	Red Tractor
**F003**[1]	M	Owner	FTF	Independent	Outdoor organic	9 *(had 400 in 2007)*	Progeny	Red Tractor, Soil Association
**F004**	M	Manager	BO	Independent	Slatted indoor	550	-	Red Tractor
**F005**	M	Owner	FTF	Independent	Straw-based indoor	0 *(had 100 in 2012)*	0 (Progeny in 2012)	None
**F006**	M	Manager	FTF	Independent	Straw-based indoor	160	Progeny	Red Tractor
**F007**	M	Owner	BO	Independent	Breeding outdoor, finishing straw-based indoor	5800	-	Red Tractor, Freedom Foods
**F008**	M	Manager	FTF on multi-site production	Company	Breeding outdoor, finishing straw-based indoor	22000	Progeny	Red Tractor, Freedom foods
**F009**	M	Manager	FTF on multi-site production	Company	Slatted indoor	4500	Progeny	Red Tractor
**F010**	M	Manager	FTF	Company	Slatted and straw-based indoor	2000	Progeny	Red Tractor
**F011**	M	Manager	FTF	Independent	Slatted indoor	150	Progeny	Red Tractor
**F012**	M	Manager	FTF	Independent	Breeding outdoor, finishing – some outdoor, some straw-based indoor	2100	Progeny	Red Tractor, Freedom Foods
**F013**	M	Owner	FTF	Independent	Breeding outdoor, finishing outdoor	30	Progeny	Quality Meat Scotland Farm Assurance Soil Association
**F014**	M	Owner	FTF	Independent	Breeding outdoor, finishing straw-based indoor	250	Progeny	SFQC Farm Assurance, SPCA Freedom Foods
**F015**	M	Manager	FTF	Independent	Straw-based indoor	130	Progeny	Red Tractor, Freedom Foods
**F016**	M	Manager	FTF	Independent	Straw-based indoor	350	Progeny	Red Tractor, Freedom Foods
**F017**	M	Manager	FO	Company	Straw-based indoor	-	300000	Red Tractor, Freedom Foods
**F018**	M	Manager	BO	Company	Outdoor	35000	-	Red Tractor, Freedom Foods
**F019**	M	Manager	BO	Company	Outdoor	40000	-	Red Tractor, Freedom Foods
**F020**	M	Owner	FO	Independent	Straw-based indoor	-	750	Red Tractor, Freedom Foods
**F021**	M	Manager	FO	Independent	Slatted indoor	-	7500	Red Tractor
**F022**	M	Manager	FTF	Independent	Slatted and straw-based indoor	230	Progeny	Red Tractor

1.4 Table S4.

Housing characteristics of questionnaire respondent farms by farm classification in UK pig farms (n=261). Respondents were asked to tick all that applied on their farm' such that for breeding to finishing pig units both breeding sows and feeding pigs would be included (n=363).

Housing characteristics and farm classification	% of respondents
**Indoor breeding**	32.98% (126)
**Breeding company**	1.31% (5)
**Outdoor breeding**	14.66% (56)
**Organic**	3.14% (12)
**Indoor feeding only**	34.55% (132)
**Outdoor feeding**	8.38% (32)

1.5 Table S5.1, S5.2, S5.3 and S5.4

Univariable analysis of farm and management characteristics and the use antimicrobials for different disease situations in different groups of pigs in the year preceding the questionnaire study.

1.5.1 Table S5.1.

Factors associated with antimicrobial use for lameness in farrowing sows in the preceding 12 months (n=115).

		No disease present	Disease present	Likelihood p-value	Odds ratio	Lower 95% CI	Upper 95% CI
**Number of sows on farm**	**Median**	105	320	**<0.001**	1.0	1.0	1.0
	**Minimum**	2	2				
	**Maximum**	40000	4000				
	**IQ range**	338	624				
**Pig density of region**	**Low**	21 (87.5%)	4 (12.5%)	0.63	Ref		
	**Moderate**	94 (75.8%)	30 (24.2%)		1.68	0.53	5.27
	**High**	85 (78.7%)	23 (21.3%)		1.42	0.44	4.55
**Pig movement type**	**Continuous**	68 (76.4%)	21 (23.6%)	**0.002**	Ref		
	**All-in-all-out**	40 (53.3%)	35 (46.7%)		2.83	1.45	5.52
**Feeding type**	**Meal/cob**	38 (64.4%)	21 (35.6%)	0.58	Ref		
	**Pellet**	69 (69.7%)	30 (30.3%)		0.79	0.40	1.56
	**Wet**	8 (57.1%)	6 (42.9%)		1.36	0.42	4.44
**Flooring type**	**Outdoor**	35 (53%)	31 (47%)	**0.003**	Ref		
	**Straw**	42 (80.8%)	10 (19.2%)		2.58	1.14	5.83
	**Slatted**	35 (74.5%)	12 (25.5%)		0.69	0.27	1.80
**Farm assured**	**No**	58 (85.3%)	10 (14.7%)	**0.046**	Ref		
	**Yes**	135 (73.7%)	48 (26.3%)		2.06	0.98	4.36
**Closed herd**	**No**	147 (77.8%)	42 (22.2%)	0.91	Ref		
	**Yes**	54 (77.1%)	16 (22.9%)		1.04	0.54	2.00
**Sows vaccination status Porcine Reproductive and Respiratory Disease Syndrome**	**No**	176 (82.2%)	38 (17.8%)	**<0.001**	Ref		
	**Yes**	27 (57.4%)	20 (42.6%)		3.43	1.75	6.75
**Sows vaccination status Erysipelas**	**No**	145 (87.9%)	20 (12.1%)	**<0.001**	Ref		
	**Yes**	58 (60.4%)	38 (39.6%)		4.75	2.55	8.84
**Sows vaccination status Influenza**	**No**	198 (79.8%)	50 (21.1%)	**0.002**	Ref		
	**Yes**	5 (38.5%)	8 (61.5%)		6.34	1.99	20.20
**Sows vaccination status Porcine Circovirus**	**No**	198 (78.9%)	53 (21.1%)	**0.049**	Ref		
	**Yes**	5 (50%)	5 (50%)		3.74	1.04	13.38
**Sows vaccination status Glässers Disease**	**No**	199 (78.3%)	55 (21.7%)	0.19	Ref		
	**Yes**	4 (57.1%)	3 (42.9%)		2.71	0.59	12.49
**Sows vaccination status Enzootic Pneumonia**	**No**	199 (77.7%)	57 (22.3%)	1	Ref		
	**Yes**	4 (80%)	1 (20%)		0.87	0.09	7.96

Table S5.2.

Factors associated with antimicrobial use for gastrointestinal disease in piglets in the preceding 12 months (n=168).

		No disease present	Disease present	Likelihood p-value	Odds ratio	Lower 95% CI	Upper 95% CI
**Number of pigs on farm**	**Median**	850	2000	**<0.001**	1.001	1.0	1.001
	**Minimum**	1	8				
	**Maximum**	73500	300000				
	**IQ range**	1823	3854				
**Pig density of region**	**Low**	14 (56%)	11 (44%)	0.35	Ref		
	**Moderate**	84 (67.7%)	40 (32.2%)		0.95	0.40	2.25
	**High**	65 (60.2%)	43 (39.8%)		1.70	0.71	4.08
**Pig movement type**	**Continuous**	48 (53.9%)	41 (46.1%)	0.15	Ref		
	**All-in-all-out**	32 (42.7%)	43 (57.3%)		1.94	1.04	3.63
**Flooring type**	**Outdoor**	33 (70.2%)	14 (29.8%)	**<0.001**	Ref		
	**Straw**	35 (67.3%)	17 (32.7%)		3.88	1.76	8.55
	**Slatted**	22 (33.3%)	44 (66.7%)		1.03	0.45	2.35
**Farm assured**	**No**	53 (77.9%)	15 (22.1%)	**0.003**	Ref		
	**Yes**	107 (58.5%)	76 (41.5%)		4.92	2.66	9.13
**Closed herd**	**No**	126 (66.7%)	63 (33.3%)	0.069	Ref		
	**Yes**	38 (54.3%)	32 (45.7%)		2.04	1.17	3.57
**Piglet E.coli vaccination status**	**No**	156 (60%)	87 (40%)	0.66	Ref		
	**Yes**	10 (58.8%)	7 (41.2%)		1.03	0.38	2.75
**Piglet Porcine Reproductive and Respiratory Syndrome vaccination status**	**No**	161 (63.6%)	92 (36.4%)	1	Ref		
	**Yes**	5 (71.4%)	2 (26.8%)		1.22	0.27	5.57
**Piglet Porcine Circovirus vaccination status**	**No**	125 (64.1%)	70 (35.9%)	0.88	Ref		
	**Yes**	41 (63.1%)	24 (36.9%)		0.92	0.53	1.62
**Piglet Enzootic Pneumonia vaccination status**	**No**	133 (62.4%)	80 (37.6%)	0.31	Ref		
	**Yes**	33 (70.2%)	14 (29.8%)		0.619	0.33	1.17
**Sows E.coli vaccination status**	**No**	140 (70%)	60 (30%)	**<0.001**	Ref		
	**Yes**	26 (42.6%)	35 (57.4%)		2.694	1.45	5.0
**Sows Clostridia vaccination status**	**No**	151 (67.4%)	73 (32.6%)	**0.002**	Ref		
	**Yes**	15 (40.5%)	22 (59.5%)		1.39	0.69	2.82
**Sows Porcine Reproductive and Respiratory Syndrome vaccination status**	**No**	156 (72.9%)	58 (27.1%)	**<0.001**	Ref		
	**Yes**	10 (21.3%)	37 (78.7%)		2.15	1.13	4.33
**Sows Porcine Circovirus vaccination status**	**No**	161 (64.1%)	90 (35.9%)	0.37	Ref		
	**Yes**	5 (50%)	5 (50%)		0.90	0.26	3.19
**Sows Enzootic Pneumonia vaccination status**	**No**	162 (63.3%)	94 (36.7%)	0.66	Ref		
	**Yes**	4 (75%)	1 (25%)		0.60	0.1	3.64
**Sows influenza vaccination status**	**No**	160 (64.5%)	88 (35.3%)	0.19	Ref		
	**Yes**	6 (46.2%)	7 (53.8%)		0.55	0.18	1.73

1.5.2 Table S5.3.

Factors associated with antimicrobial use for respiratory disease in feeding pigs in the preceding 12 months (n=310).

		No disease present	Disease present	Likelihood p-value	Odds ratio	Lower 95% CI	Upper 95% CI
**Number of pigs on farm**	**Median**<br>**Minimum**<br>**Maximum**<br>**IQ range**	300<br>1<br>73500<br>1425	1990<br>16<br>300000<br>3114	**<0.001**	1.0	1.0	1.0
**Pig density of region**	**Low**	13 (52%)	12 (48%)	0.081	Ref		
	**Moderate**	66 (53.2%)	58 (46.8%)		0.588	0.25	1.41
	**High**	42 (38.9%)	66 (61.1%)		0.56	0.33	0.94
**Pig movement type**	**Continuous**	59 (49.2%)	61 (50.8%)	0.089	Ref		
	**All-in-all-out**	39 (37.9%)	64 (32.1%)		1.59	0.93	2.71
**Feeding type**	**Meal/cob**	14 (40%)	21 (60%)	0.069	Ref		
	**Pellet**	86 (52.8%)	77 (47.2%)		0.58	0.17	1.98
	**Wet**	5 (27.8%)	13 (72.2%)		0.34	0.12	1.01
**Flooring type**	**Outdoor**	22 (68.8%)	10 (31.2%)	**0.01**	Ref		
	**Straw**	65 (53.3%)	57 (46.7%)		4.13	1.58	10.80
	**Slatted**	16 (34.8%)	30 (65.2%)		1.93	0.84	4.42
**Ventilation type**	**Outdoor**	23 (69.7%)	10 (30.3%)	**0.009**	Ref		
	**Natural**	80 (42.8%)	107 (57.2%)		0.25	0.81	0.74
	**Artificial**	9 (36%)	16 (64%)		0.75	0.32	1.80
**Farm assured**	**No**	50 (73.5%)	18 (26.5%)	**<0.001**	Ref		
	**Yes**	66 (36.1%)	117 (63.9%)		0.50	2.66	9.13
**Closed herd**	**No**	80 (42.3%)	109 (57.7%)	**0.011**	Ref		
	**Yes**	42 (60%)	28 (40%)		2.04	1.17	3.57
**Source of weaners**	**Multiple**	13 (39.4%)	20 (60.6%)	0.98	Ref		
	**Single**	38 (39.2%)	59 (60.8%)		1.01	0.45	2.27
**Porcine Reproductive and Respiratory Syndrome vaccination status**	**No**	123 (47.9%)	134 (52.1%)	0.62	Ref		
	**Yes**	1 (25%)	3 (75%)		0.36	0.04	3.54
**Porcine Circovirus vaccination status**	**No**	115 (50%)	115 (50%)	0.026			
	**Yes**	9 (29%)	22 (71%)		0.4`	0.18	0.93
**Enzootic Pneumonia vaccination status**	**No**	119 (50.6%)	116 (49.4%)	**0.002**	Ref		
	**Yes**	5 (19.2%)	21 (80.8%)		0.23	0.09	0.64
**Actinobacillus Pleuropneumonia vaccination status**	**No**	135 (52.1%)	135 (52.1%)	0.45	Ref		
	**Yes**	2 (100%)	2 (100%)		0	0	0

1.5.3 Table S5.4.

Factors associated with antimicrobial use for lameness in dry sows in the preceding 12 months (n=121).

		No disease present	Disease present	Likelihood p-value	Odds ratio	Lower 95% CI	Upper 95% CI
**Number of pigs on farm**	**Median**	40	285	**<0.001**	1.0	1.0	1.0
	**Minimum**	2	10				
	**Maximum**	40000	7000				
	**IQ range**	270	630				
**Pig density of region**	**Low**	19 (76%)	6 (24%)	0.44	Ref		
	**Moderate**	78 (62.9%)	46 (37.1%)		1.87	0.696	5.013
	**High**	70 (64.8%)	38 (35.2%)		1.72	0.633	4.669
**Pig movement type**	**Continuous**	63 (45.7%)	75 (54.3%)	0.96	Ref		
	**All-in-all-out**	9 (45%)	11 (55%)		1.03	0.4	2.635
**Feeding type**	**Meal/cob**	30 (47.6%)	33 (52.4%)	0.84	Ref		
	**Pellet**	44 (48.9%)	46 (51.1%)		0.95	0.499	1.81
	**Wet**	7 (41.2%)	10 (58.8%)		1.30	0.499	3.844
**Flooring type***	**Outdoor**	33 (64.7%)	18 (35.3%)	**0.015**	Ref		
	**Straw**	50 (42%)	69 (58%)		1.83	0.24	14.133
	**Slatted**	2 (50%)	2 (50%)		2.53	1.28	4.994
**Farm assured**	**No**	57 (83.8%)	11 (16.2%)	**<0.001**	Ref		
	**Yes**	105 (53.4%)	78 (46.6%)		3.85	1.90	7.82
**Closed herd**	**No**	127 (67.2%)	62 (32.8%)	0.2	Ref		
	**Yes**	41 (58.6%)	29 (41.4%)		1.45	0.82	2.547
**Sows vaccination status Porcine Reproductive and Respiratory Disease Syndrome**	**No**	157 (73.4%)	57 (26.6%)	**<0.001**	Ref		
	**Yes**	13 (27.7%)	34 (72.3%)		7.20	3.55	14.614
**Sows vaccination status Erysipelas**	**No**	137 (83%)	28 (17%)	**<0.001**	Ref		
	**Yes**	33 (34.4%)	63 (65.6%)		9.34	5.20	16.772
**Sows vaccination status Influenza**	**No**	168 (67.7%)	80 (32.3%)	**<0.001**	Ref		
	**Yes**	2 (15.4%)	11 (84.6%)		11.55	2.50	53.339
**Sows vaccination status Porcine Circovirus**	**No**	168 (66.9%)	83 (33.1%)	**0.004**	Ref		
	**Yes**	2 (20%)	8 (80%)		8.10	1.68	38.98
**Sows vaccination status Glässers Disease**	**No**	168 (66.1%)	86 (33.9%)	0.052	Ref		
	**Yes**	2 (28.6%)	5 (71.4%)		4.89	0.93	25.692
**Sows vaccination status Enzootic Pneumonia**	**No**	166 (64.9%)	90 (35.1%)	0.66	Ref		
	**Yes**	4 (80%)	1 (20%)		0.46	0.05	4.188

2 Supplementary Figures

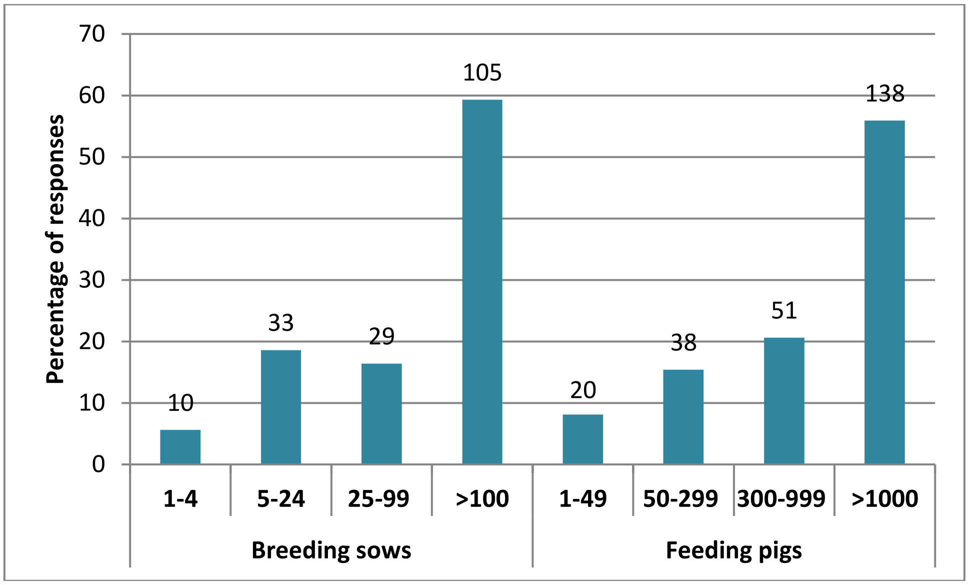

2.1 Figure S1

Number of breeding sows and feeding pigs on the farms of questionnaire respondents in a study on antimicrobial use in UK pig production.
